# Mechanical Properties and Eco-Efficiency of Steel Fiber Reinforced Alkali-Activated Slag Concrete

**DOI:** 10.3390/ma8115383

**Published:** 2015-10-30

**Authors:** Sun-Woo Kim, Seok-Joon Jang, Dae-Hyun Kang, Kyung-Lim Ahn, Hyun-Do Yun

**Affiliations:** 1 Department of Construction Engineering Education, Chungnam National University, Daejeon 34134, Korea; sw.kim@cnu.ac.kr (S.-W.K.); 2 Department of Architectural Engineering, Chungnam National University, Daejeon 34134, Korea; suk-joon@nate.com (S.-J.J.); davidkang88@gmail.com (D.-H.K); ahnkl08@gmail.com (K.-L.A)

**Keywords:** alkali-activated slag (AAS), mechanical performance, eco-efficiency, ordinary Portland cement (OPC), sustainable binder

## Abstract

Conventional concrete production that uses ordinary Portland cement (OPC) as a binder seems unsustainable due to its high energy consumption, natural resource exhaustion and huge carbon dioxide (CO_2_) emissions. To transform the conventional process of concrete production to a more sustainable process, the replacement of high energy-consumptive PC with new binders such as fly ash and alkali-activated slag (AAS) from available industrial by-products has been recognized as an alternative. This paper investigates the effect of curing conditions and steel fiber inclusion on the compressive and flexural performance of AAS concrete with a specified compressive strength of 40 MPa to evaluate the feasibility of AAS concrete as an alternative to normal concrete for CO_2_ emission reduction in the concrete industry. Their performances are compared with reference concrete produced using OPC. The eco-efficiency of AAS use for concrete production was also evaluated by binder intensity and CO_2_ intensity based on the test results and literature data. Test results show that it is possible to produce AAS concrete with compressive and flexural performances comparable to conventional concrete. Wet-curing and steel fiber inclusion improve the mechanical performance of AAS concrete. Also, the utilization of AAS as a sustainable binder can lead to significant CO_2_ emissions reduction and resources and energy conservation in the concrete industry.

## 1. Introduction

Concrete is the most widely used construction material and uses a great amount of cement. Furthermore, anthropogenic CO_2_ emissions due to the production of cement is rapidly increasing with the increase of urban development. In order to address this, alkali-activated slag (AAS) has been considered as an alternative binder to cement, as well as a way of reusing available industrial by-products. Damineli *et al.* [1] proposed two indicators which allow measuring the eco-efficiency of cement use, binder intensity and CO_2_ intensity. Both indicators were tested using two sets of data from literature, one Brazilian and the other from 28 different countries. Yang *et al.* [2] derived the relationship between the CO_2_ and binder intensities of different concretes from a regression analysis of a comprehensive database in Korea. However, their applicability was hindered because the AAS concretes obtained with sodium silicate activators exhibit higher drying shrinkage rates than in ordinary Portland cement (OPC). At present, fiber inclusion is commonly accepted as the way to improve shrinkage behavior of AAS concrete [3,4]. In addition to above, many researches have been conducted to improve or enhance the performance of AAS concrete by incorporation of fibers [5,6,7]. The primary purposes of the fiber inclusion are to control cracking and to increase the fracture toughness of the cement matrix by a bridging action that is controlled by debonding, sliding and pulling-out the reinforcing fibers during both micro and macro-cracking of the matrix. Bernal *et al.* [5] concluded that alkali-activated slag concrete reinforced with steel fibers shows three times higher flexural toughness than Portland cement concretes at early ages of curing. Aydin and Baradan [6] reported that alkali-activated slag/silica fume mortars present significantly higher mechanical performance than OPC based mortar at the same fiber dosage due to the higher bonding properties between fiber and alkali-activated slag/silica fume mortar interfacial zone compared to OPC mortar. In relation to the use of fibers and development of sustainable materials, the structural behaviors of timber beams have also been studied [8,9,10].

The aim of this study is to improve the mechanical performance of Ca(OH)_2_-based AAS concrete by incorporation of steel fibers because it has been found from other previous research results that microfiber such as polyethylene (PE) or polyvinyl alcohol (PVA) is useful for improving tensile performance of a cementitious composite without coarse aggregate. In this study, concrete was mixed with crushed granite, and therefore steel fibers that have high tensile performance and larger diameter compared to the microfiber were used for mixing the concrete. The effects of incorporation of steel fibers on the compressive and flexural performance of AAS concretes with the main variables being steel fiber volume fraction and curing condition are investigated in this paper. The test results are compared to the OPC concrete specimens, with a view to identifying their performances and potential applications as construction materials. In this study, an alternative fiber-reinforced concrete to OPC concrete with a specified compressive strength value of 40 MPa was produced by using 30 mm length hooked-end steel fibers. The utilization of AAS in concrete will be helpful in reducing environmental problems and greenhouse gas emissions associated with the Portland cement production, and in conserving existing natural resources.

## 2. Experimental Section

### 2.1. Materials and Specimen Preparation

Ground granulated blast-furnace slag (GGBS) and Type I OPC were used as binders for AAS concrete and ordinary concrete, respectively. The chemical compositions of the GGBS and OPC used in this study are given in Table 1. The specific surfaces for the OPC and the GGBS were 325 and 430 m^2^/kg, respectively. The GGBS has a 21.2 μm maximum particle size and an 8.5 μm average particle size. To produce the AAS concrete, AAS binder was produced by the activation of GGBS with calcium hydroxide as the primary activator. Sodium silicate (Na_2_SiO_3_) and sodium carbonate (Na_2_CO_3_) were used as auxiliary activators. The selection of primary and auxiliary activators was based on studies previously conducted in Korea [2,11,12]. From the chemical composition of GGBS presented in the table, the basicity coefficient (K_b_) and hydration modulus (HM) of GGBS were calculated to be 0.92 and 1.68, respectively.

**Figure 1:**
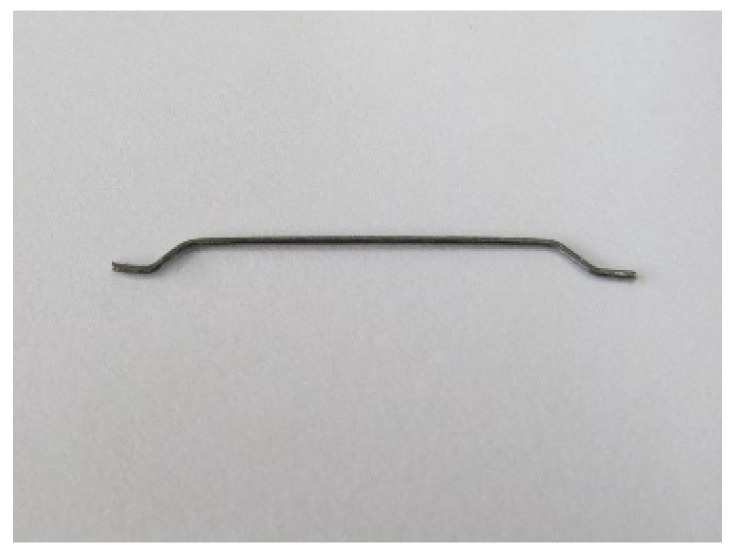
Shape of a hooked-end steel fiber.

Locally available river sand (maximum particle size of 5 mm) and crushed granite (maximum particle size of 20 mm) were used as fine and coarse aggregates, respectively. The results of sieve analysis for fine and coarse aggregates met the continuous standard curves specified in KS F 2526 [13]. As listed in Table 2, OPC and AAS concretes were prepared with a water-to-binder ratio of 0.55 and a sand-to-coarse aggregate ratio of 0.45. Hooked-end steel fibers shown in Figure 1 were made from mild carbon steel with a tensile strength of 1100 MPa. The fibers were 30 mm long and 0.5 mm in diameter, giving an aspect ratio of 60. The percentage of the steel fiber added ranged from 0%–2.0% by weight of the binder as seen in Table 3.

To produce OPC and AAS concretes, the binder and the aggregate were initially dry-mixed for a minute. After the dry-mixing, water including a superplasticizer was added and the time for mixing was planned to be long enough to prevent any segregation in concrete. The required quantities of steel fibers were then added separately in small amounts to avoid fiber balling. The freshly mixed steel fiber-reinforced concrete was poured in two layers into cylindrical (Φ100 mm × 200 mm) and prismatic (100 mm × 100 mm × 400 mm) steel moulds for compressive and flexural tests, respectively. For each mix, nine cylinders (three cylinders at each age) and three prisms were cast in steel moulds and kept in a mist room at 23 °C and 95% relative humidity for 24 h until demoulding. After demoulding, specimens for wet-curing were preserved in water at 23 °C and the other specimens for dry-curing were placed in air at 23 ± 5 °C and 50 ± 5% relative humidity until 1 day before testing. For all mixes, 225 and 75 specimens were made and tested for compressive and flexural properties, respectively.

**Table 1:** Chemical composition (% by mass) of GGBS and OPC.

Component	GGBS	OPC
Silicon dioxide (SiO_2_)	34.7	20.9
Aluminium oxide (Al_2_O_3_)	13.8	5.39
Calcium oxide (CaO)	40.1	64.7
Iron oxide (Fe_2_O_3_)	0.11	2.38
Magnesium oxide (MgO)	4.38	1.51
Titanium dioxide (TiO_2_)	0.74	1.33
Sodium oxide (Na_2_O)	0.20	0.27
Potassium oxide (K_2_O)	0.48	0.22
Sulfur trioxide (SO_3_)	4.83	1.65
Loss on ignition (LOI)	2.70	5.80
Basicity coefficient (K_b_)	0.92	2.52
Hydration modulus (HM)	1.68	3.43

Notes: K_b_ = (CaO + MgO)/(SiO_2_ + Al_2_O_3_); HM = (CaO + MgO + Al_2_O_3_)/SiO_2_.

**Table 2:** Mixture proportions of OPC and AAS concretes.

Mix	w/b	S/a	Unit Weight (kg/m^3^)				
			W	C	AAS	S	G
OPC	0.55	0.45	205	373	-	756	924
AAS	0.55	0.45	205	-	373	756	924

Notes: w/b is water-to-binder ratio; S/a is sand-to-aggregate ratio; W is water; C is cement; S is sand; and G is coarse aggregate.

**Table 3:** Variables for mechanical tests.

Test	Specimen	*V_f_* (%)	Curing Method
Compression	OPC	0.0, 0.5, 1.0, 1.5, 2.0	Wet-curing
	AAS-dry	0.0, 0.5, 1.0, 1.5, 2.0	Dry-curing
	AAS-wet	0.0, 0.5, 1.0, 1.5, 2.0	Wet-curing
Flexure	OPC	0.0, 0.5, 1.0, 1.5, 2.0	Wet-curing
	AAS-wet	0.0, 0.5, 1.0, 1.5, 2.0	Wet-curing

Notes: *V_f_* is steel fiber volume fraction.

### 2.2. Test Methods

The cross-sectional area of each cylinder for compressive test was calculated using an average of three diameter measurements taken in two intersecting directions at the mid-height of the specimen. At 3, 7, and 28 days after concrete casting, compressive strength tests of the cylindrical specimens were performed. The cylinders were tested in compression as per ASTM C 39 [14] until failure. The flexural strength tests were conducted at 28 days in accordance with ASTM C 78 [15].

## 3. Results and Discussion

### 3.1. Compressive Performance

**Figure 2:**
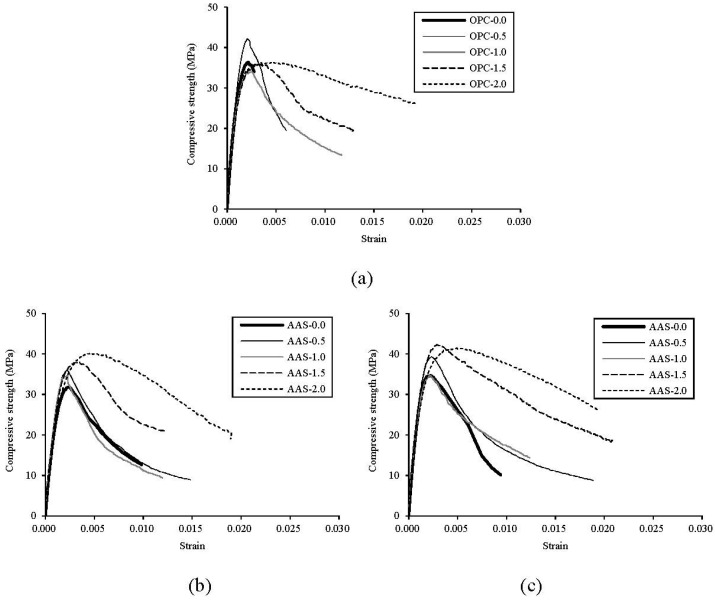
Stress-strain curves of test specimens at 28 days. (**a**) OPC-wet; (**b**) AAS-dry; (**c**) AAS-wet.

Compressive strength *versus* strain curves of OPC, AAS-dry, and AAS-wet specimens at 28 days with different fiber contents are presented in Figure 2. The average curves were drawn from the three test results for each mix. The error in the measurement was calculated as the standard deviation of the compressive strength from three samples. The maximum standard deviations in the compressive strength were 1.56, 2.45, and 3.05 MPa at 3, 7, and 28 days, respectively. As seen in Figure 2a, the descending curve of OPC-0.0 specimen has almost vanished due to brittle failure right after peak stress, whereas the other OPC specimens with fiber and all of the AAS specimens have various descending curves as seen in Figure 2a,b. As the steel fiber volume fraction increases, the curve after peak stress descended slowly. It can be inferred that the compressive failure mode of concrete changed from a brittle to a more ductile failure due to the steel fiber inclusion. The effect of steel fibers on the compressive strength of AAS concrete is more noticeable in case of higher fiber inclusion [5]. Karunanithi and Anandan (2014) reported that the increase of steel fiber inclusion improved the compressive strength of AAS concrete [16]. As shown in Figure 2b,c, AAS-wet specimens exhibit more stable post behavior than AAS-dry specimens. It reflects that water-curing enhances the bond properties in the interfacial zone between fibers and the matrix phase for AAS binders compared to dry-curing.

**Figure 3:**
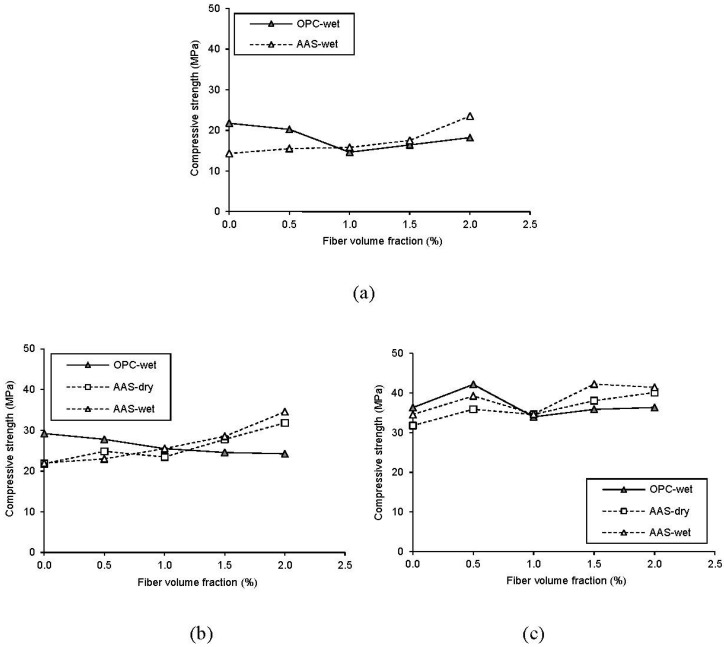
Compressive strength development with age (**a**) 3 days; (**b**) 7 days; (**c**) 28 days.

The effects of fiber inclusion on the compressive strengths with age are presented in Figure 3. Until 7 days, OPC-0.5 specimen shows a little less strength than OPC-0.0. At 28 days, however, OPC-0.5 specimens show higher compressive strength value than OPC-0.0. For OPC specimens with over 1.0% of fiber volume fraction, there is no noticeable increase in the compressive strength compared to OPC-0.0 specimen. It is thought that the steel fibers were not effectively dispersed in concrete when mixing. However, compressive strength values of AAS specimens have significantly increased by fiber inclusion. At 28 days, the addition of steel fibers in AAS concrete has a significant effect on the enhancement of the compressive strength by 1.9%–17.8%. In addition to the fiber inclusion, the compressive strength development of concrete is affected by curing methods; it is clear that water-curing is more efficient for the strength development of concrete than dry-curing as shown in Figure 3b,c.

**Figure 4:**
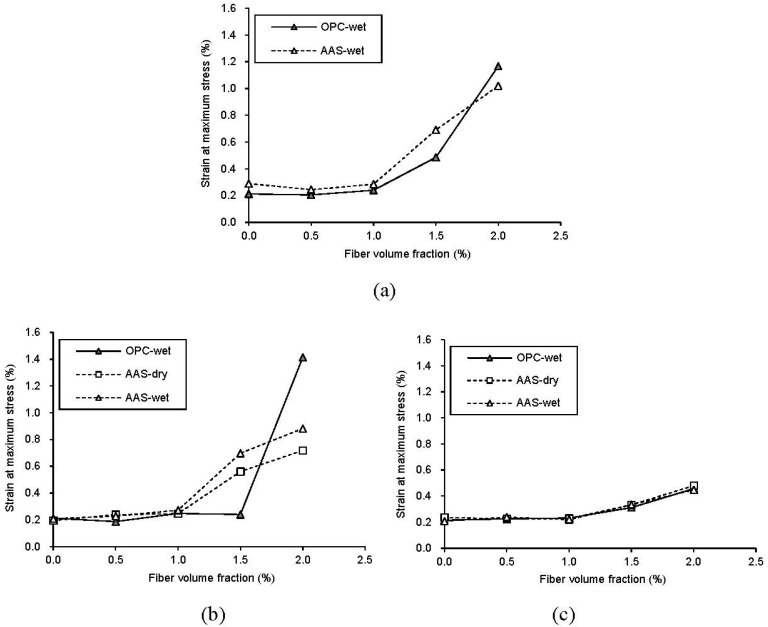
Variation of compressive strain at maximum stress with age. (**a**) 3 days; (**b**) 7 days; (**c**) 28 days.

Figure 4 shows the effect of fiber inclusion on the strain at maximum stress of concrete at each age. As shown in the figure, the strain value has a tendency to decrease with the age of concrete as expected. From the results for strain at each age, except OPC specimens at 7 days, all specimens show a similar trend in the strain increase ratio, so it can be inferred that the effect of binder type on the strain capacity of concrete is negligible. For curing method, the increase ratio on the strain of AAS concrete is similar between dry- and wet-curing. For strain values at 7 days, regardless of curing methods, the AAS-1.5 specimen showed a noticeably higher strain than OPC. The compressive test results are summarized in Table 4.

**Table 4:** Compressive test results.

Specimens	Compressive Strength (MPa)					Strain at Max. Compressive Strength (%)				
	3 Days	7 Days		28 Days		3 Days	7 Days		28 Days	
	Wet	Dry	Wet	Dry	Wet	Wet	Dry	Wet	Dry	Wet
OPC-0.0	21.7	-	29.2	-	36.3	0.212	-	0.211	-	0.214
OPC-0.5	20.2	-	27.8	-	42.2	0.205	-	0.186	-	0.225
OPC-1.0	14.6	-	25.5	-	34.0	0.241	-	0.247	-	0.231
OPC-1.5	16.4	-	24.5	-	35.9	0.486	-	0.239	-	0.311
OPC-2.0	18.2	-	24.2	-	36.4	1.167	-	1.413	-	0.457
AAS-0.0	14.3	21.8	22.0	31.8	34.6	0.290	0.195	0.209	0.236	0.207
AAS-0.5	15.5	24.8	23.0	35.9	39.3	0.245	0.238	0.227	0.225	0.238
AAS-1.0	15.8	23.4	25.5	34.6	34.7	0.285	0.246	0.272	0.228	0.219
AAS-1.5	17.5	27.8	28.5	38.1	42.3	0.692	0.561	0.697	0.333	0.334
AAS-2.0	23.5	31.8	34.6	40.1	41.4	1.020	0.719	0.883	0.480	0.447

**Figure 5:**
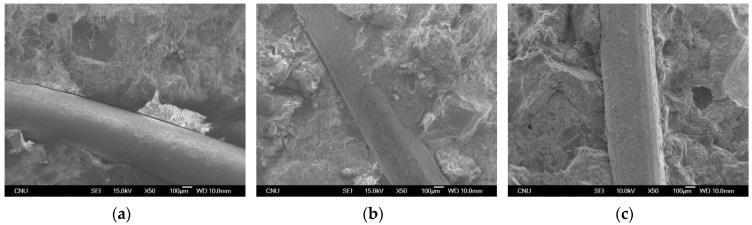
SEM micrographs of steel fiber–matrix transition zone. (**a**) OPC-wet; (**b**) AAS-dry; (**c**) AAS-wet.

The compressive strength values of AAS concrete were significantly increased by fiber inclusion whereas OPC concrete shows no noticeable change in the compressive strength. It can be noted that lateral confinement by steel fiber is improved due to the smaller particle size of GGBS compared to OPC. Furthermore, the increase ratio in compressive strength for AAS concrete with wet-curing is higher than that with dry-curing. This indicates that wet-curing enhances the bonding properties between fiber and mortar phase for AAS based binders. Figure 5 shows SEM micrographs of the steel fiber–matrix transition zone. From the micrographs in the figure, it is clear that using AAS binder turns the surface of the steel fiber from smooth to coarse. The SEM micrographs also confirmed that the bond property in the interfacial zone between steel fibers and matrix phase for AAS binders is better than that for OPC based binders. These enhanced bond characteristics of alkali activated binders have also been reported in other research results [17,18]. Shi and Xie [17] also reported that the formation of the dense and uniform transition zone in the Na_2_SiO_3_^−^ activated slag mortars can be attributed to several factors such as the water reducing function of Na_2_SiO_3_ and the high initial concentration of [SiO_4_]^4−^ in the pore solution.

### 3.2. Flexural Performance

**Figure 6:**
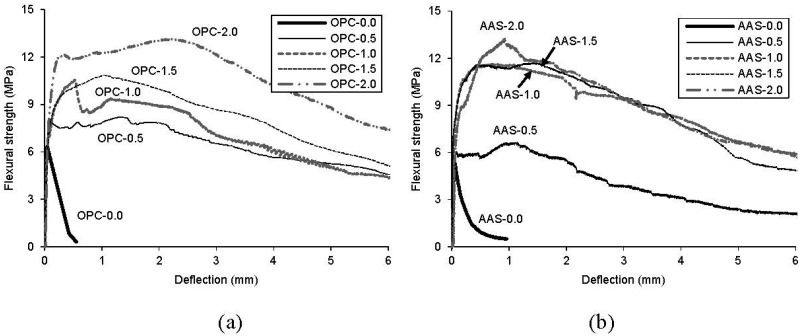
Flexural strength-deflection curves of test specimens at 28 days. (**a**) OPC specimens; (**b**) AAS specimens.

A flexural strength-deflection curve itself may be quite sensitive in distinguishing amongst different fiber inclusions, and the curve roughly indicates differences in the toughness performance of concrete with different fiber volume fractions. Figure 6 shows flexural strength-deflection curves for all specimens. All the curves are the average values for three specimens. As expected, the ultimate strength is higher for concrete with more fiber volume fractions, due to bridging action. However, there was no significant effect of binder type or fiber inclusion on the first cracking strengths. The ultimate strength was noticeably improved when the fiber volume fraction was above 1.0%. In particular, AAS-1.0, AAS-1.5 and AAS-2.0 specimens showed very similar flexural behavior as shown in Figure 6a, whereas the ultimate strengths of AAS-0.0 and AAS-0.5 specimens were lower than those of OPC-0.0 and OPC-0.5 specimens due to lower compressive strengths. In Table 5, first crack strength (f_f_), first crack deflection (δ_f_), ultimate strength (f_f_), and deflection at the peak load (δ_f_) are given. As shown in flexural responses in Figure 6, the descending portion of the curve is steeper when the ultimate strength is higher. Therefore, it can be inferred that the residual strength beyond the peak load decreases faster so that the post-crack toughness changes.

**Table 5:** Strengths and deflections at both first crack and peak load.

Specimen	First Crack		Peak Load	
	f_f_ (MPa)	δ_f_ (mm)	f_u_ (MPa)	δ_u_ (mm)
OPC-0.0	6.329	0.049	6.329	0.049
OPC-0.5	6.577	0.047	8.197	1.303
OPC-1.0	5.755	0.042	10.557	0.531
OPC-1.5	5.064	0.047	10.855	1.035
OPC-2.0	6.646	0.055	13.130	2.221
AAS-0.0	5.501	0.050	5.501	0.050
AAS-0.5	4.674	0.044	6.638	1.145
AAS-1.0	5.249	0.029	11.615	0.705
AAS-1.5	5.910	0.019	11.714	1.427
AAS-2.0	4.180	0.047	13.262	0.931

ASTM C1018 [19] is the most common method used for evaluating the flexural toughness of FRC (fiber reinforced concrete). In this study, the ASTM flexural toughness indices and the ASTM residual strength factors are adopted for calculating the post-crack toughness of test specimens. The ASTM specifies the flexural toughness indices as I_5_ (3δ), I_10_ (5.5δ), I_20_ (10.5δ), I_30_ (15.5δ), and I_50_ (25.5δ). For the residual strength factors, R_5,10_, R_10,20_, R_20,30_ and R_30,50_ are also specified in the ASTM. In this paper, for the flexural toughness indices and residual strength factors, I_100_ (50δ), I_200_ (100δ), R_50,100_, and R_100,200_ were added to consider overall flexural response including the deflection at peak load of test specimens. For the R_50,100_ and R_100,200_ added, the factors were reduced as per the following equations:

R_50,100_ = 2 × (I_100_ − I_50_) (1)

R_100,200_ = 2 × (I_200_ − I_100_) (2)

**Figure 7:**
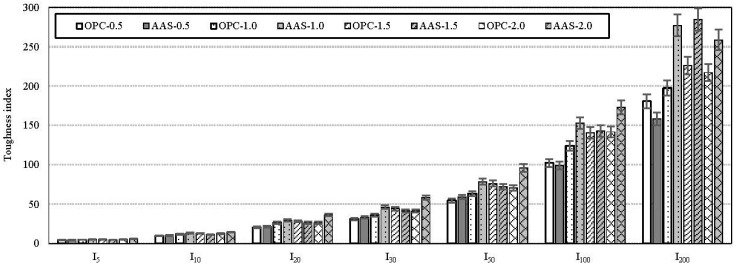
Comparison of toughness indices.

Figure 7 shows the effect of binder type on the ASTM toughness indices for all specimens. All are average values for three specimens. It can be seen that the ASTM toughness indices are particularly sensitive in distinguishing amongst different fiber volume fractions, which is more obvious when the fiber volume fraction is higher than 1.0%. AAS specimens exhibit similar or higher toughness index than OPC specimens, regardless of fiber volume fraction. In particular, for R_50,100_ and R_100,200_ which are toughness indices added in this paper, the difference of indices between AAS and OPC specimens was widened. This means that the AAS binder is more efficient for bonding in the interfacial zone between steel fibers and the matrix phase than OPC binder during post-cracking behavior.

**Figure 8:**
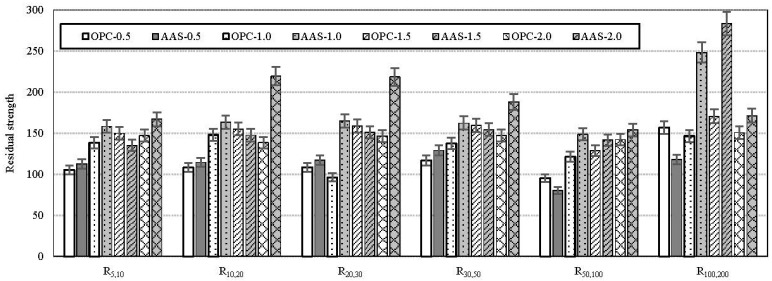
Comparison of residual strength factors.

Figure 8 shows the effect of binder type on the ASTM residual strength factors for all specimens. As shown in the figure, AAS binder could be better for residual strength of FRC than OPC binder. It can be thought that the internal stress distribution in the tension zone would be enhanced due to the formation of the dense and uniform transition zone in the matrix as seen in Figure 5.

### 3.3. Eco-Efficiency of Alkali-Activated Slag

Korea LCI Database Information Network [20] and Japanese database [21] were referred to in order to evaluate the CO_2_ emissions of concrete. Table 6 and Table 7 show CO_2_ evaluation of the OPC and the AAS concrete, respectively. As listed in the tables, the CO_2_ emission of the AAS concrete is 15%~24% of that of the OPC concrete because the AAS based binder has about 97% lower CO_2_ emission than the OPC based binder. The CO_2_ emissions of concretes considering fiber inclusion are compared in Table 8.

To compare environmental efficiency, a binder intensity (*bi*) and CO_2_ intensity (*ci*) were proposed by Damineli BL *et al.* [1]. It was reported that the binder intensity yields the efficiency of using clinker and other hard to find clinker substitutes. The CO_2_ intensity permits an estimation of the mix design’s global warming potential. The indices are calculated as:

(3) $$bi=\frac{b}{p}$$

(4) $$ci=\frac{c}{p}$$

where *b* is the total consumption of binder materials (kg/m^3^), *c* is the total CO_2_ (kg/m^3^) emitted to produce, and *p* is the compressive strength (MPa) at 28 days.

**Table 6:** CO_2_ evaluation of OPC concrete.

Item	Material and Production		
	A	B	A·B
	kg/m^3^	CO_2_-kg/kg	CO_2_-kg/m^3^
OPC	373	0.944	352.1
Sand	756	0.0026	2.0
Coarse	924	0.0075	6.9
Water	205	1.96 × 10^−4^	0.0
Admixture	0.1492	0.25	0.0
Conc. production	2258	0.008	18.1
**Sum**	–	–	**379.2**
Wet-curing	–	–	**38.5**
Total * = 417.7 CO_2_-kg/m^3^			

Note: * CO_2_ emission of steel fiber (1.6 CO_2_-kg/kg) is not included.

**Table 7:** CO_2_ evaluation of AAS concrete.

Item	Material and Production		
	A	B	A·B
	kg/m^3^	CO_2_-kg/kg	CO_2_-kg/m^3^
GGBS	373	0.0265	9.9
Sand	756	0.0026	2.0
Coarse	924	0.0075	6.9
Water	205	1.96 × 10^−4^	0.0
Admixture	0.1492	0.25	0.0
Conc. production	2258	0.008	18.1
**Sum**	–	–	**36.9**
Dry-curing	–	–	**0.0**
Wet-curing	–	–	**38.5**
Total * = 36.9 CO_2_-kg/m^3^ for AAS-dry; **75.4 CO_2_-kg/m^3^ for AAS-wet**			

Note: * CO_2_ emission of steel fiber (1.6 CO_2_-kg/kg) is not included.

**Table 8:** Final CO_2_ emission of concrete with fiber inclusion.

Mix	Curing Method	CO_2_ Emission with Fiber Volume Fractions (CO_2_-kg/kg)				
		0.0%	0.5%	1.0%	1.5%	2.0%
OPC	wet	417.7	420.6	423.6	426.6	429.6
AAS	dry	36.9	39.9	42.9	45.9	48.9
AAS	wet	75.4	78.4	81.4	84.4	87.4

**Figure 9:**
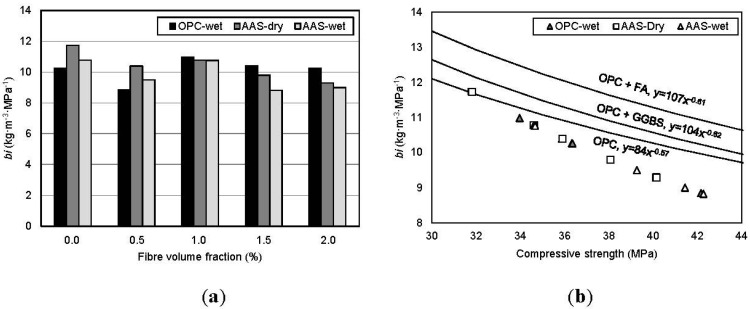
Comparison of binder intensity (*bi*). (**a**) fiber volume fraction; (**b**) compressive strength.

The binder intensity with variables and the relationship between the binder intensity and compressive strength are presented in Figure 9. As seen in Figure 9a, it is clear that the binder intensity can be reduced by wet-curing and fiber inclusion. In case of concrete with less than 1.0% of fiber volume fraction, OPC specimens have lower binder intensity compared to AAS specimens. However, as fiber volume fraction increases, the binder intensities of AAS specimens are lower than those of OPC specimens. It can be inferred that AAS binder can enhance performance, especially the compressive strength of concrete, and reduce the total amount of binder necessary to achieve the performance required. The best-fit curves in previous research [2] are adopted to compare the binder intensity in this study and are presented in Figure 9b. As shown in the figure, the binder intensity calculated in this study is below the best-fit curve of OPC (Yang *et al.*, 2013). AAS concrete has lower binder intensity compared to OPC concrete even though it was reported that Ca(OH)_2_-based AA GGBS concrete requires a greater amount of binder in order to obtain the same compressive strength as OPC concrete [2].

**Figure 10:**
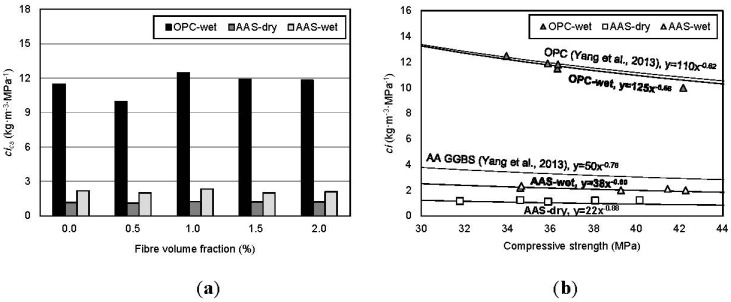
Comparison of CO_2_ intensity (*ci*). (**a**) Fiber volume fraction; (**b**) compressive strength.

Figure 10 shows the CO_2_ intensity with variables and the relationship between the CO_2_ intensity and compressive strength. As estimated in Table 8, the CO_2_ intensity of AAS concrete is about 20%–25% of that of OPC concrete. In view of the curing method, as shown in Figure 10a, wet-curing increases the CO_2_ intensity by 40%–50% compared to dry-curing because electric equipment is needed to maintain the water at a constant temperature for curing. The best-fit curves in previous research [2] are adopted to compare the CO_2_ intensity in this study and are presented in Figure 10b. As shown in the figure, it is indicated that the estimated CO_2_ intensity for OPC is around the best-fit curve. However, there is little difference between the curves because Yang *et al.* [2] considered CO_2_ emitted in production and transport as *c*. In this study, CO_2_ emitted for transport was omitted because the CO_2_ emission is influenced by the transportation distance between the plant and construction sites. In this study, CO_2_ emitted in production only (cradle to gate) was considered to calculate CO_2_ intensity (*ci*), and the best-fit curves for AAS-wet and -dry lie under the curve proposed by Yang *et al.* [2].

## 4. Conclusions

In this study, AAS was used as an alternative binder to OPC in order to reduce environmental impact of concrete. To estimate eco-efficiency of AAS based binder, compressive tests were conducted and the effects of fiber inclusion and wet-curing on the two indicators (*bi* and *ci*) were evaluated. The following observations and conclusions can be made and drawn on the basis of the compressive test results and intensity estimation in this study.

1. AAS based binders may provide similar compressive performance with a lower fiber content due to better fiber dispersion and enhanced bond characteristics in the steel fiber–matrix transition zone. Furthermore, the wet-curing method is very helpful for the performance enhancement of AAS with steel fibers.
2. It can be inferred that AAS binder can enhance performance, especially the compressive strength of concrete, and can reduce the total amount of binder necessary to achieve the performance required.
3. Based on the results of the ASTM toughness index and residual strength factor, it is concluded that AAS binder is more efficient for bonding in the interfacial zone between steel fibers and matrix phase than OPC binder in post-cracking behavior. This is because the internal stress distribution in the tension zone would be enhanced due to the formation of the dense and uniform transition zone in the matrix.
4. The significant effects of fiber inclusion and wet-curing on the eco-efficiency of AAS were confirmed on the basis of the binder intensity and CO_2_ intensity. It indicates that more economic and eco-efficient fiber reinforced composites with AAS can be produced. In the future, additional studies on the mechanical properties of AAS concrete under fatigue and creep are needed.
